# A case of VEXAS syndrome presenting with unusual bone marrow granulomas: a diagnostic dilemma

**DOI:** 10.1186/s41927-023-00343-w

**Published:** 2023-07-10

**Authors:** Khiem T. Vu, Rachel M. Wolfe, Jonathan E. Lambird, Danielle L. V. Maracaja

**Affiliations:** Atrium Health Wake Forest Baptist Medical Center, Winston-Salem, NC 27157 USA

**Keywords:** VEXAS, UBA1, Granulomas, Vacuoles, Autoinflammatory, Case report

## Abstract

**Background:**

VEXAS is a recently described inflammatory disease caused by mutations in the *UBA1* gene. Symptoms are diverse and include fevers, cartilaginous inflammation, lung inflammation, vasculitis, neutrophilic dermatoses, and macrocytic anemia. Cytoplasmic inclusions in myeloid and erythroid progenitors in the bone marrow are a hallmark feature. Here we report the first case of VEXAS with non-caseating granulomas in the bone marrow.

**Case presentation:**

A 62-year-old Asian male presented with fevers, erythema nodosum, inflammatory arthritis, and periorbital inflammation. Labs were significant for persistently elevated inflammatory markers and macrocytic anemia. Over the years his symptoms and inflammatory markers only improved with glucocorticoids and recurred when prednisone dose was lowered below 15–20 mg daily. He underwent bone marrow biopsy showing non-caseating granulomas and PET scan showing hilar/mediastinal lymphadenopathy. He was initially diagnosed with IgG4-related disease (treated with rituximab) and later sarcoidosis (treated with infliximab). After failing these agents, the possibility of VEXAS was considered and later confirmed by molecular testing.

**Conclusions:**

To the best of our knowledge, this is the first observation of non-caseating granulomas in VEXAS, a cautionary reminder of its non-specificity since misinterpretation can lead to diagnostic delay. VEXAS should be in the differential in patients with symptoms of chronic inflammation responding positively to steroids (but not to B-cell depletion or TNF inhibition), which is in line with previous literature.

## Background

The recent discovery of somatic, inactivating mutations in *UBA1* (ubiquitin-activating enzyme E1) is an important defining genetic abnormality in patients presenting with a myriad of systemic inflammatory symptoms and hematologic abnormalities [1, 2]. First reported in 2020, these mutations defined a new disease referred to as VEXAS (vacuoles in myeloid precursors, E1 enzyme, X-linked, autoinflammatory, somatic mutation) [1]. Patients, who are men above 40 years old, develop symptoms of systemic inflammation that include fevers, ear and nose cartilage inflammation, lung inflammation, vasculitis, and skin disease. Patients may even fulfill diagnostic criteria for overlapping diseases like giant cell arteritis, relapsing polychondritis, and Sweet syndrome [3]. Hematological manifestations are a prominent feature of VEXAS, which include macrocytic anemia, cytopenias, and increased risk of myelodysplastic syndrome, plasma cell disorders, and progressive bone marrow failure [3, 4]. Sustained remission has not been obtained with standard disease modifying agents. Unfortunately, patients often must rely on long courses of prednisone to control symptoms [5, 6].

This case report describes a patient who presented with systemic inflammatory symptoms and hematologic abnormalities prior to the identification of VEXAS syndrome [1]. The presence of bone marrow granulomas, a finding not previously reported in VEXAS, led to delay in the patient’s diagnosis and management. Therefore, reporting this potential histologic finding in bone marrow evaluation is pertinent for accurate diagnosis and appropriate management.

## Case presentation

In late summer of 2019, a 62-year-old Asian male with chronic inactive hepatitis B presented to the hospital for 1–2 weeks of left periorbital pain and swelling. Brain MRI showed findings of left preseptal and orbital cellulitis. He was empirically treated and discharged with intravenous ceftriaxone and vancomycin and symptoms improved.

During that hospital stay, however, additional labs showed positive antinuclear antibody (ANA) with a titer of 1:320 (speckled pattern), erythrocyte sedimentation rate (ESR) of 97 mm/hr (reference range 0–33 mm/hr), C-reactive protein (CRP) of 169 mg/L (reference range < 5 mg/L), and mildly elevated immunoglobulin subclass G4 (IgG4) of 282 mg/dL (reference range 2.4–121.0 mg/dL). Antineutrophil cytoplasmic antibodies (ANCA) were negative and complement C3 and C4 levels were normal. Due to concern for autoimmune etiologies, he was evaluated by our rheumatology consult service and found to have synovitis at the left third proximal interphalangeal (PIP) and right first metacarpophalangeal (MCP) joints. But the patient recovered well and no steroids nor further immunosuppression were recommended at the time. Subsequent outpatient labs were negative for antibodies against double stranded DNA (dsDNA) and Smith.

Two months later however, the patient developed fevers and shortness of breath. He was admitted for hypoxia and computed tomography angiography (CTA) of the chest showed diffuse alveolar infiltrates. He was empirically started on high-dose prednisone, starting dose 60 mg daily, with significant clinical improvement. Although his diagnosis remained unclear, he established care with outpatient rheumatology where his prednisone dose was tapered over the next eight months. Clinically, he continued to feel well. Nevertheless, multiple blood tests during follow-up appointments revealed a persistently elevated serum IgG4 in the 240–270 mg/dL range. By comparison, serum IgG1 was 1100 mg/dL (reference range 341–894 mg/dL), IgG2 was 844 mg/dL (reference range 171–632 mg/dL), and IgG3 was 56.4 mg/dL (reference range 18.4–106.0 mg/dL). ESR and CRP remained at 66 to ≥ 140 mm/hr and 20–90 mg/L, respectively, and ferritin was elevated at 790 ng/mL (reference range 30–300 ng/mL). He was also noted to have persistent macrocytic anemia with mean corpuscular volume (MCV) ranging 102–128 fL (reference range 80–96 fL). Evaluation for vitamin B12 and folate deficiency, alcohol use, and liver disease was negative. Macrocytosis was attributed to his long-term use of tenofovir for hepatitis B.

A few days after the patient tapered prednisone to 7.5 mg daily, his fevers not only returned, but he also developed tender erythema nodosum in his extremities and bilateral periorbital edema. He started azathioprine as a steroid-sparing agent but continued to have a predictable episodic pattern of symptom recurrence whenever prednisone was tapered below 10 mg daily, and brisk improvement after prednisone was increased.

During a particularly severe episode in November 2020, he was admitted and found to have new-onset leukopenia (white blood cells 4,000/µL; reference range 4,400 − 11,000/µL) and thrombocytopenia (platelets 8,100/µL; reference range 150,000-450,000/µL). Hemoglobin was 12.1 g/dL (reference range 14.0-17.5 g/dL) and MCV was 108 fL. Reticulocyte index was calculated to be 0.54% (< 2%, consistent with decreased red blood cell production). He underwent fluorodeoxyglucose (FDG)-positron emission tomography (PET) scan that showed diffuse hypermetabolic uptake in the bone marrow. Bone marrow trephine core biopsy and clot section were performed and showed hypercellularity (approximately 80% cellular) with myeloid predominance and erythroid hypoplasia, increased percentage of plasma cells (approximately 10% cellularity), lymphohistiocytic aggregates, and non-caseating granulomas. Erythroid and myeloid lineages showed no dysplasia. Megakaryocytes showed a range of morphologic findings with occasional hypolobated forms. Bone marrow flow cytometry revealed a minute lambda restricted monotypic plasma cell and a small atypical CD4 dim, T-cell population of unknown etiology. Grocott’s methenamine silver stain (GMS) and acid-fast bacillus (AFB) stains were performed and were negative for fungal or acid-fast organisms.

Although his serum free kappa and lambda light chains were found to be elevated (65 mg/L and 83 mg/L, respectively) and urine protein electrophoresis (UPEP) showed monoclonal lambda (elevated at 408.8 mg/L), the serum protein electrophoresis (SPEP) showed no monoclonal spike, other than an elevated gamma globulin ranging 2.0-2.7 g/dL (reference range 0.5–1.6 g/dL) on repeat measurements. Immunofixation electrophoresis (IFE) was polyclonal, even on repeat measurements over the next few years. Subsequent UPEPs did not reproduce the previously detected monoclonal protein. Over the next year, the patient continued to depend on prednisone doses ≥ 10 mg daily to control breakthrough fevers and erythema nodosum, and associated increases in his already elevated ESR and CRP.

In December 2021, considering his initial manifestations of periorbital/orbital inflammation and elevated baseline serum IgG4, azathioprine was stopped and he was started on rituximab for possible IgG4-related disease. After two infusions of rituximab 1000 mg, symptoms continued to recur. Further rituximab infusions were discontinued.

The patient underwent a second PET scan due to the continued lack of diagnosis and suspicion of malignancy. It again showed diffuse hypermetabolic uptake in the bone marrow, but newly hypermetabolic mediastinal and right hilar lymph nodes. Subsequent bronchoscopy showed multiple white, inflammatory bronchial lesions on an erythematous base, but negative infectious studies on bronchoalveolar washings. Considering the previously detected non-caseating granulomas in his bone marrow and these recent findings, we started infliximab 3 mg/kg for presumed sarcoidosis, two years from initial presentation. The patient however had no clinical improvement after six months and infliximab was discontinued after five doses.

During follow-up appointment in early 2023, the patient continued to report fevers while on prednisone 10 mg daily. He was found to have recurrent erythema nodosum, bilateral periorbital edema, active synovitis at the right third PIP joint, and worsening ESR and CRP.

**Fig. 1:**
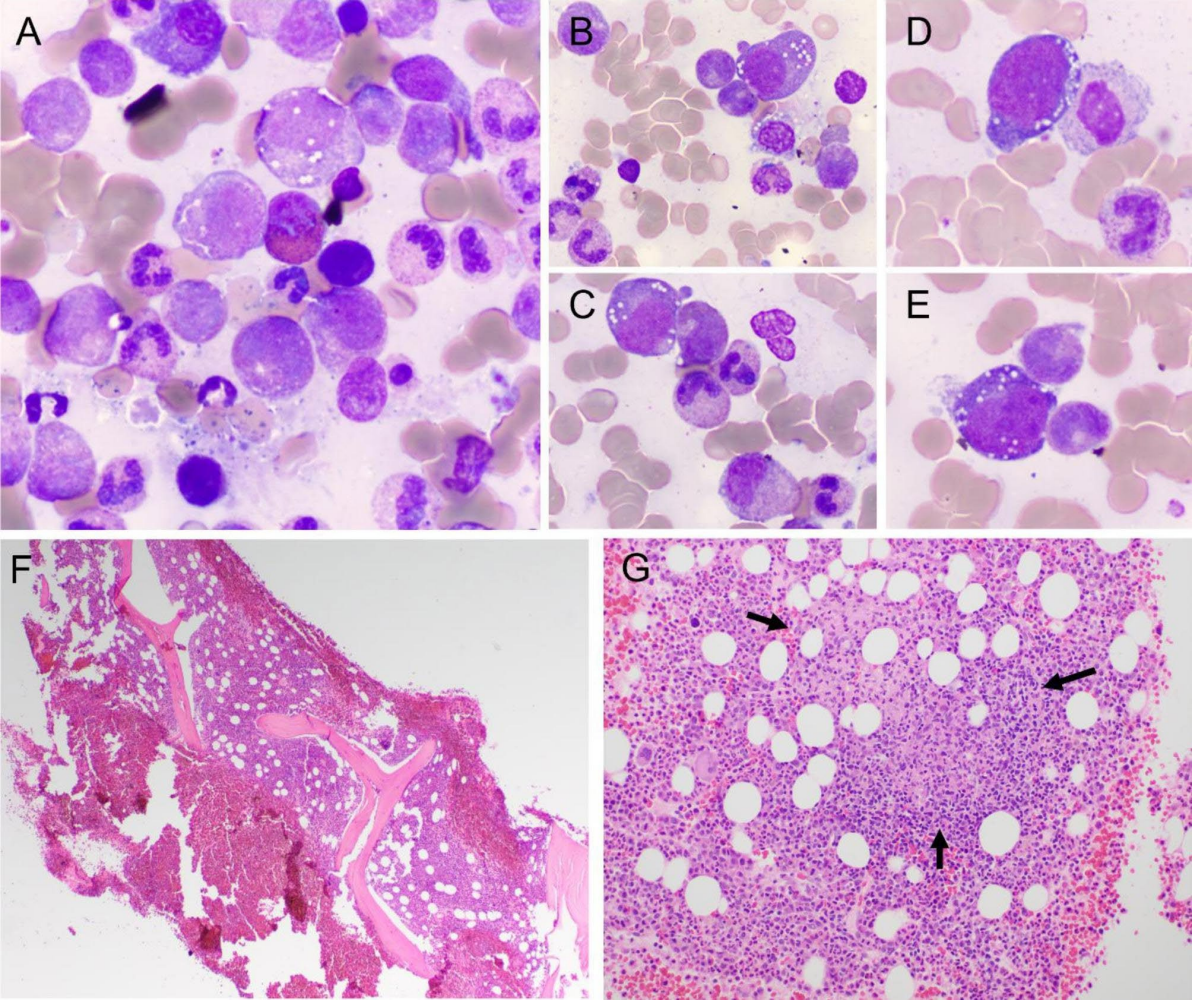
The Wright-Giemsa stain showed a spicular and cellular marrow with myeloid predominance and no overt dysplasia. There was vacuolization in the myeloid precursors (A-C, 100x oil) and erythroid precursors (D-E, 100x oil). F shows H&E stain in a hypercellular marrow for age (approximately 80% cellular) with scattered granulomas and lymphohistiocytic infiltrates in the trephine core biopsy (4x) and in the clot section (black arrows; 20x)

Given the patient’s treatment failures to azathioprine, rituximab, and infliximab, no evidence of malignancy, and negative infectious workup, the possibility of VEXAS syndrome was considered. He had several compatible features: hypermetabolic bone marrow, macrocytic anemia, inflammatory arthritis, inflammatory lung disease, periorbital edema, erythema nodosum, and symptom relief with steroids. His previous bone marrow biopsy was reexamined and observed to contain myeloid and erythroid precursors with cytoplasmic vacuoles. Megakaryocytes showed occasional monolobated forms with rare eccentrically placed nuclei (Fig. 1). Copper levels and serologies for *Coxiella burnetti* infection were obtained to rule out alternative etiologies for cytoplasmic vacuoles and previously noted bone marrow granulomas, respectively. A peripheral blood sample was sent to ARUP Laboratories for next-generation sequencing (Myeloid Malignancies Mutation Panel). He was found to have a missense mutation in *UBA1* (c.122T > C, pMet41Thr) with a variant allele frequency of 53%. Two other missense mutations *DNMT3A* (c1979A > G, pTyr660Cys) with variant allele frequency 27.3% and *ASXL1* (c.3376 C > T, pHis1126Tyr) with variant allele frequency of 46.2% were noted. The *DNMT3A* is considered a variant of known clinical significance and the *ASXL1* mutation is considered a tier 2 mutation (variant of unknown clinical significance). Hence, the diagnosis of VEXAS syndrome was made, about 3.5 years after his disease first presented.

## Discussion and conclusions

VEXAS syndrome is an adult-onset, autoinflammatory disease first described in 2020 by Beck et al. [1]. It is caused by acquired somatic mutations in the X-linked *UBA1* gene and has an estimated prevalence of 1 in 4269 males and 1 in 26,238 females older than 50 years [7]. *UBA1* is essential in protein ubiquitination and defects in this process are thought to promote dysregulated innate immunity and systemic inflammation. Patients are almost always male due to the *UBA1* gene’s location on the X chromosome, and symptoms typically present at age > 55 years. Clinical features are diverse, affect multiple organs, and contain aspects of rheumatologic and hematologic disease. They include systemic symptoms, ocular inflammation, chondritis, inflammatory arthritis, vasculitis, lung inflammation, neutrophilic dermatoses, blood clots, cytopenias, and macrocytic anemia. Furthermore, a particularly important feature in these patients is a positive response to steroids.

A characteristic hallmark of VEXAS is the presence of cytoplasmic vacuoles in myeloid and erythroid precursors in the bone marrow, although secondary causes like chronic alcoholism, copper deficiency, and myelodysplasia should be excluded [5].

The presumptive diagnosis of IgG4-RD in our patient was not made lightly. Our patient’s CRP levels were markedly elevated, which is unusual for IgG4-RD in the absence of concurrent aortitis or infection. Moreover, we only had elevated serum IgG4 to rely on and no tissue biopsy demonstrating IgG4-positive cells, lymphoplasmacytic infiltrate, and/or storiform fibrosis. Unfortunately, there was no further tissue to biopsy and even PET scan at that time did not reveal other avenues to secure a tissue diagnosis. Given his significant sequela of long-term glucocorticoid use, a steroid-sparing agent was indicated. Since IgG4-RD can cause similar symptoms and there was not a better alternative diagnosis considered at the time, we elected to treat him with rituximab knowing that it covers IgG4-RD as well as many other rheumatic diseases. Although we hoped for a robust response to rituximab, a negative response would serve as another diagnostic clue to his disease.

Only after our patient’s failure to a trial of infliximab of adequate duration did we initiate work-up for VEXAS, which was confirmed by the detection of a *UBA1* mutation by targeted myeloid mutational analysis by next generation sequencing. The most common mutations involve methionine 41 in exon 3 of *UBA1* (Met31Thr, Met41Val, Met41Leu), although additional mutations involving serine 56 and exon 3 splice site were reported recently [2].

It is still to be determined the causative relationship of the several histologic findings in the bone marrow biopsy of VEXAS patients. Some of the most common initially described bone marrow findings include hypercellularity, myeloid predominance, erythroid hypoplasia, cytoplasmic vacuolization in erythroid and myeloid precursors, and dysplasia [8]. Recently, Olteanu et al. presented the morphologic findings of 94 cases of VEXAS syndrome and found the previously described findings in addition to abnormal findings in the megakaryocytic lineage. The megakaryocytic abnormalities included large forms with condensed pink cytoplasm (“pink bellies”), nuclear fragmentation, eccentrically placed nuclei and monolobation [9]. In our patient, the cytoplasmic vacuolization was not initially noted, but upon review of the histologic findings 3.5 years later, we could find the vacuolization in more than 5% of the myeloid and erythroid precursors. There was no dysplasia, but the megakaryocytes showed a range of morphology with occasional monolobation and rare eccentric placed nuclei.

Our case was unique in that non-caseating granulomas and lymphohistiocytic infiltrates were also detected in the bone marrow trephine core biopsy and clot section which, although nonspecific, raised concern for sarcoidosis given our patient’s symptoms of inflammatory arthritis, erythema nodosum, and ocular disease [10]. The presence of granulomas has not been previously reported in VEXAS, to the best of our knowledge. Granulomas consist of clusters of macrophages which occur in response to a pathogen, foreign body, or other immune response [11]. The granulomas in infectious etiologies are caused by an attempt to contain the antigen. However, the cause of granuloma formation in non-infectious conditions is still to be determined and may be the result of immune-mediated activation [11]. Beck et al. described how dysfunction in *UBA1* in myeloid cells causes defects in ubiquitylation, leading to activation of unfolded protein and stress response pathways [1]. One can hypothesize that monocytic activation and dysregulation could have triggered an immune response to form granulomas in this case. Although this hypothesis cannot be completely confirmed, it is important to collect these cases to further identify possible pathophysiologic mechanisms for granulomatous formation in VEXAS syndrome.

About 50% of VEXAS syndrome patients can develop myelodysplastic syndrome (MDS) so co-management between rheumatology and hematology/oncology is invaluable [12]. There is also a risk for plasma cell neoplasms [8]. Our patient was found to have a small lambda monotypic plasma cell population on bone marrow flow cytometry, which was not confirmed on follow up UPEP, SPEP, and IFE. In addition, we found an additional pathogenic mutation in DNA methyltransferase 3 A (*DNMT3A*). The enzyme encoded by *DNMT3A* regulates gene expression. It functions by methylating DNA at CpG sites. The missense mutation in this patient causes loss of function in this enzyme and affects all lines in hematopoiesis. *DNMT3A* is commonly associated with clonal hematopoiesis [13]. *ASXL1* is frequently mutated in MDS. However, the mutation noted in this patient has not been previously described in myeloid malignancy and it is considered a variant of unknown significance (tier 2). Mutations in epigenetic regulators occur with increasing frequency with age and are associated with poorer prognosis in MDS [14–16]. Although our patient did not have clear evidence of an overt hematologic malignancy, these additional findings are consistent with a co-existing, clonal hematopoiesis of indeterminate potential (CHIP), requiring vigilant monitoring for development of future myeloid malignancy [4, 17].

Another interesting finding was the presence of a distinct abnormal CD4-positive T-cell population in this case. Obiorah et al. previously described abnormalities in the flow cytometry related to increased T-cell activation and cytotoxic immunophenotype [18]. Our case did not show a cytotoxic immunophenotype, but an abnormal CD4 dim population. CD4-positive cells are important in mediating immune response against pathogens and in autoimmunity. Since this increased abnormal population has not been described in VEXAS, it is difficult to know the real cause of this finding. However, we speculate the increase may have occurred in response to the patient’s autoinflammatory state at the time of the bone marrow biopsy.

Since VEXAS was only recently described, effective treatments are still being investigated.

Sustained remission is refractory to multiple traditional immunosuppressives like B-cell depletion and TNF-alpha inhibition as seen in our patient [2, 6]. As such, glucocorticoids remain the primary treatment to control symptoms. As knowledge and recognition of this disease grows, more therapeutic options are emerging. Encouraging data shows benefits for tocilizumab, ruxolitinib, and azacytidine [6, 19]. A clinical trial is underway at the NIH investigating hematopoietic stem cell transplant, which would in theory, be curative [20].

In summary, this was a case with a protracted clinical course with multiple lines of therapy and unusual, atypical histologic findings of granulomas in the bone marrow, complicating the diagnosis and treatment. Furthermore, since VEXAS syndrome is a newly described entity, it is imperative to continue reporting and documenting unusual or unexpected findings in affected patients.

## Data Availability

Laboratory, imaging, and pathology data are presented in this article. Additional data not presented in this article is available from the corresponding author on reasonable request.

## Abbreviations

ANCA: antineutrophil cytoplasmic antibodies
ANA: antinuclear antibody
CHIP: clonal hematopoiesis of indeterminate potential
CRP: c-reactive protein
CTA: computed tomography angiography
dsDNA: double stranded DNA
ESR: erythrocyte sedimentation rate
IEOP/IFE: immunoelectrophoresis/immunofixation
IgG4: immunoglobulin subclass G4
MCP: metacarpophalangeal
NIH: National Institutes of Health
PET: positron emission tomography
PIP: proximal interphalangeal
SPEP: serum protein electrophoresis
TNF: tumor necrosis factor
*UBA1*: ubiquitin-activating enzyme E1
UPEP: urine protein electrophoresis
VEXAS: vacuoles in myeloid precursors, E1 enzyme, X-linked, autoinflammatory, somatic mutation
